# Beyond Bronchiolitis Obliterans: In-Depth Histopathologic Characterization of Bronchiolitis Obliterans Syndrome after Lung Transplantation

**DOI:** 10.3390/jcm11010111

**Published:** 2021-12-27

**Authors:** Arno Vanstapel, Stijn E. Verleden, Eric K. Verbeken, Peter Braubach, Tinne Goos, Laurens De Sadeleer, Janne Kaes, Bart M. Vanaudenaerde, Danny Jonigk, Maximilian Ackermann, Laurens J. Ceulemans, Dirk E. Van Raemdonck, Arne P. Neyrinck, Robin Vos, Geert M. Verleden, Birgit Weynand, on behalf of the Leuven Lung Transplant Group

**Affiliations:** 1 Department of Chronic Diseases and Metabolism, BREATHE, Katholieke Universiteit Leuven, 3000 Leuven, Belgium; arno.vanstapel@kuleuven.be (A.V.); stijn.verleden@uantwerpen.be (S.E.V.); tinne.goos@kuleuven.be (T.G.); laurens.desadeleer@kuleuven.be (L.D.S.); janne.kaes@kuleuven.be (J.K.); bart.vanaudenaerde@kuleuven.be (B.M.V.); laurens.ceulemans@uzleuven.be (L.J.C.); dirk.vanraemdonck@uzleuven.be (D.E.V.R.); robin.vos@uzleuven.be (R.V.); geert.verleden@uzleuven.be (G.M.V.); 2 Department of Pathology, University Hospitals Leuven, 3000 Leuven, Belgium; erik.verbeken@kuleuven.be; 3 Antwerp Surgical Training, Anatomy and Research Centre (ASTARC), University of Antwerp (UA), 2610 Wilrijk, Belgium; 4 Department of Thoracic & Vascular Surgery, University Hospital Antwerp (UZA), 2650 Edegem, Belgium; 5 Department of Pneumology, University Hospital Antwerp (UZA), 2650 Edegem, Belgium; 6 Biomedical Research in Endstage and Obstructive Lung Disease Hannover (BREATH), Hannover Medical School, 30625 Hannover, Germany; Braubach.Peter@mh-hannover.de (P.B.); Jonigk.Danny@mh-hannover.de (D.J.); 7 Department of Respiratory Diseases, University Hospitals Leuven, 3000 Leuven, Belgium; 8 Institute of Pathology and Department of Molecular Pathology, Helios University Clinic Wuppertal, University of Witten–Herdecke, 58455 Witten, Germany; maximilian.ackermann@uni-mainz.de; 9 Institute of Functional and Clinical Anatomy, University Medical Center of the Johannes Gutenberg University Mainz, 55131 Mainz, Germany; 10 Department of Thoracic Surgery, University Hospitals Leuven, 3000 Leuven, Belgium; 11 Department of Cardiovascular Sciences, Katholieke Universiteit Leuven, 3000 Leuven, Belgium; arne.neyrinck@uzleuven.be; 12 Department of Anesthesiology, University Hospitals Leuven, 3000 Leuven, Belgium

**Keywords:** bronchiolitis obliterans syndrome, BOS, chronic lung allograft dysfunction, CLAD, BO, histology, histopathology

## Abstract

Bronchiolitis obliterans syndrome (BOS) is considered an airway-centered disease, with bronchiolitis obliterans (BO) as pathologic hallmark. However, the histologic spectrum of pure clinical BOS remains poorly characterized. We provide the first in-depth histopathologic description of well-characterized BOS patients and patients without chronic lung allograft dysfunction (CLAD), defined according to the recent consensus guidelines. Explant lung tissue from 52 clinically-defined BOS and 26 non-CLAD patients (collected 1993–2018) was analyzed for histologic parameters, including but not limited to airway lesions, vasculopathy and fibrosis. In BOS, BO lesions were evident in 38 (73%) patients and varied from concentric sub-epithelial fibrotic BO to inflammatory BO, while 10/14 patients without BO displayed ‘vanishing airways’, defined by a discordance between arteries and airways. Chronic vascular abnormalities were detected in 22 (42%) patients. Ashcroft fibrosis scores revealed a median of 43% (IQR: 23–69) of normal lung parenchyma per patient; 26% (IQR: 18–37) of minimal alveolar fibrous thickening; and 11% (IQR: 4–18) of moderate alveolar thickening without architectural damage. Patchy areas of definite fibrotic damage to the lung structure (i.e., Ashcroft score ≥5) were present in 28 (54%) patients. Fibrosis was classified as bronchocentric (*n* = 21/28, 75%), paraseptal (*n* = 17/28, 61%) and subpleural (*n* = 15/28, 54%). In non-CLAD patients, BO lesions were absent, chronic vascular abnormalities present in 1 (4%) patient and mean Ashcroft scores were significantly lower compared to BOS (*p* = 0.0038) with 78% (IQR: 64–88) normally preserved lung parenchyma. BOS explant lungs revealed evidence of various histopathologic findings, including vasculopathy and fibrotic changes, which may contribute to the pathophysiology of BOS.

## 1. Introduction

Long-term survival after lung transplantation is limited by chronic lung allograft dysfunction (CLAD), affecting >50% of patients within 5 years after transplantation [1,2]. CLAD is clinically defined by a persistent decline in forced expiratory volume in one second (FEV1) of ≥20% compared to baseline, without another identifiable cause. Currently, there are two main clinical phenotypes recognized [3]. Bronchiolitis obliterans syndrome (BOS) is characterized by an obstructive pulmonary function deficit and air trapping on computed tomography (CT) [3]. In 2011, restrictive allograft syndrome (RAS) was first described and later defined by a restrictive pulmonary function deficit and persistent radiological CT opacities [4,5].

BOS is commonly referred to as an airway-centered disease, characterized by bronchiolitis obliterans (BO). BO was first defined outside the context of lung transplantation (i.e., after viral infection and inhalation of toxic fumes) as a fibrosing inflammatory process involving small bronchioles [6]. Subsequent studies described BO in lung transplantation and BO became the hallmark of BOS [7,8,9,10]. Histologic descriptions of BOS reported various pathologic lesions ranging from airway lesions to vascular abnormalities and fibrotic changes [11,12]. The largest histological study on CLAD explant lungs hitherto was performed on 25 explant lungs by Saggar et al. [12]. Interestingly, they reported pulmonary arteriopathy and venopathy in approximately 80% of investigated lungs. In addition, Martinu and colleagues examined 12 explant lungs from CLAD patients and found varying fibrosis and inflammation in BO, and several other lesions (e.g., cholesterol clefts, vascular injury) [11].

All currently available histopathologic descriptions of BOS actually precede the 2014 and 2019 diagnostic CLAD consensus guidelines [3,5,13]. Therefore, these historical histological reports are no longer consistent with the current diagnostic work-up, and furthermore precede the first clinical definition of RAS. In fact, previous histopathologic descriptions of BOS likely even included potential RAS patients, as both restrictive lung function changes [10] and radiologic evidence of fibrosis/pleural thickening [11] were described in some included patients. The histomorphological spectrum of 21 RAS lungs was recently described by our group, demonstrating large heterogeneity, ranging from pleuroparenchymal fibro-elastosis and non-specific interstitial pneumonia to irregular fibrosis-induced subpleural/paraseptal emphysema, and BO lesions in the majority of RAS patients [14]. The histological spectrum and diversity within BOS, however, remains ill-defined.

In addition to defining the histological spectrum of CLAD, a better understanding of the histopathologic changes in stable lung transplant patients is essential to better discriminate between histopathologic changes due to CLAD and non-CLAD related histological changes that may occur after lung transplantation. However, due to inherent limitations in collecting samples of non-CLAD patients, literature remains scarce. Assessment of endobronchial biopsies in stable lung transplant patients revealed thickening of the basal airway membrane and increased mononuclear airway inflammation in stable lung transplant patients, compared to non-transplant controls [15,16]. However, remarkably, to our knowledge, no reports are currently available describing histopathologic changes in explant tissue of lung transplant patients who died without CLAD. Nevertheless, many histological parameters (e.g., fibrotic changes, vasculopathy) can only be accurately assessed in explant lungs.

In the current study, we aim to provide the first in-depth histopathologic characterization of BOS patients, well-defined according to the most recent CLAD diagnostic consensus guidelines and compare findings to patients who died without CLAD. We hypothesize that, besides the BO lesion, other important histopathologic features may be present, and that clinical BOS might present differently on histopathological examination.

## 2. Materials and Methods

### 2.1. Patient Selection and BOS Diagnosis

**Figure 1:**
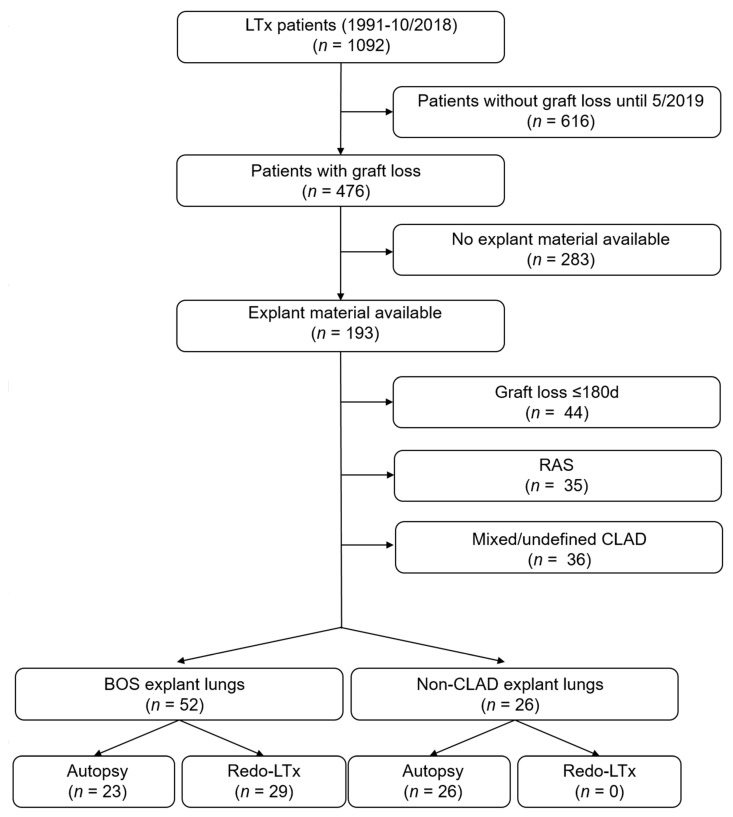
Flowchart of patient selection. LTx: lung transplantation; BOS: bronchiolitis obliterans syndrome; RAS: restrictive allograft syndrome.

We retrospectively selected patients with a clinical BOS diagnosis, and available explant lung tissue, obtained at autopsy or redo-transplantation. All patients who underwent lung transplantation at University Hospitals Leuven (Leuven, Belgium) between January 1991 and October 2018 (follow-up until 1 May 2019) were eligible for inclusion (Figure 1). Graft loss was defined as death or redo-transplantation, and clinical characteristics of all patients were extracted from clinical chart review. CLAD diagnosis and staging was performed according to the recent International Society of Heart and Lung Transplantation (ISHLT) consensus guidelines [3,5]. All transplant patients were enrolled in a standard follow-up protocol with routine follow-up visits at our center, as previously reported. BOS was defined by a persistent (>3 months) FEV_1_ decline of ≥20% compared to the mean of the two best post-operative FEV_1_ measurements obtained >3 weeks apart, in absence of another cause of pulmonary function decline [3]. Patients with RAS, mixed (both after initial BOS diagnosis and RAS diagnosis) or undefined CLAD phenotype were excluded based on the presence of a restrictive lung function deficit (i.e., >10% decline in TLC or, if TLC data was unavailable, a >20% drop in forced vital capacity (FVC)) and the presence of persistent radiological opacities [3,5]. Time-of-onset of CLAD (BOS) grade 1 was defined as the time of the initial persistent FEV1 decline of 20–35%, grade 2 as the onset of FEV1 decline of 35–50%, grade 3 as a decline of 50–65%, and grade 4 as a FEV1 decline of ≥65% [3]. Patients with definite clinical BOS until graft loss and patients without CLAD at graft loss (i.e., <20% FEV1 decline) were included. Patients with RAS, mixed CLAD (both after initial BOS diagnosis and RAS diagnosis) or undefined CLAD phenotype were excluded. Patients with <180 days graft survival were also excluded because definite BOS was not expected. 

### 2.2. Sample Collection and Histopathologic Assessment

All explant tissue was routinely obtained and processed for diagnostic histopathologic examination. This included formalin fixation, macroscopic grossing with at least one biopsy from each lobe, paraffin embedding, 5 µm sectioning and staining with hematoxylin and eosin. All hematoxylin and eosin staining’s (median 5 slides/patient, IQR [4,5,6], range [2,3,4,5,6,7,8,9,10]) and available additional staining’s (e.g., Elastica Van Gieson, Trichrome Masson, staining’s for micro-organisms) from the included patients’ explant lungs were retrieved from the lab archives. Slides were digitalized using a digital pathology slide scanner (Philips IntelliSite Ultra Fast Scanner, Best, The Netherlands). Systematic histopathologic examination, using a scoring grid, was performed by two experienced lung pathologists together, until consensus was reached (Table S1). Presence of airway-centered lesions was reviewed by assessing BO, lymphocytic bronchiolitis (B-grade rejection [17,18]), large-airway lymphocytic bronchitis (E-grade [19]), bronchiectasis and squamous metaplasia. Vanishing airways were defined by presence of ≥3 larger arteries (vessel diameter > 500 µm) without accompanying airway (BO lesions were counted as presence of an airway) in a single biopsy. Vascular lesions included presence of acute rejection (A-grade rejection [18]), pulmonary arteriopathy as evidenced by intimal hyperplasia and/or media hypertrophy, venopathy evidenced by intimal hyperplasia or venous occlusion and presence of bronchial artery vasculopathy. In the alveolar compartment, presence of emphysema, intra-alveolar foamy macrophages, cholesterol clefts, intra-alveolar fibrin, organizing pneumonia and acute fibrinous and organizing pneumonia was assessed. Fibrotic changes were quantified using the Ashcroft score by a single observer [20]. Ashcroft scores were determined based on visual assessment of 2 mm [2] fields on three hematoxylin and eosin-stained slides from three distinct locations. The Ashcroft scoring technique is further elaborated in the supplementary methods and illustrated in Figure S1. If an Ashcroft score of ≥5 was noted in at least one field (i.e., presence of increased fibrosis with definite damage to the lung structure), this was additionally reviewed by two lung pathologists and the general pattern of fibrosis was non-mutually exclusively categorized (i.e., bronchocentric, paraseptal and subpleural). The mean Ashcroft score was calculated for each patient to assess the overall extent of fibrotic remodeling. 

### 2.3. Statistical Analysis and Ethics

Patient characteristics and histopathologic findings were compared using Fisher Exact test and Mann Whitney test. GraphPad statistical software (Prism, version 7.01, La Jolla, CA, USA) was used for all analyses. A *p* value of <0.05 was considered significant, Bonferroni-Dunn post-hoc testing was applied for comparison of histopathologic findings. This study was approved by the local Ethics Committee (S52174). 

## 3. Results 

### 3.1. Patient Characteristics

In total, 52 patients with clinical BOS and available explant lung tissue were included. For 29 (56%) patients, explant tissue was available from redo-transplantation, whereas for 23 (44%) patients, explant tissue was obtained at autopsy. Patient characteristics of BOS patients are summarized in Table 1. Briefly, redo-transplant patients were younger compared to autopsy patients (*p* = 0.0077), had more cystic fibrosis and bronchiectasis as underlying disease (*p* = 0.0003) and underwent more bilateral lung transplant procedures (*p* = 0.016). In addition, baseline FEV_1_ and forced vital capacity (FVC) was higher in redo transplant patients (*p* = 0.0012 and *p* = 0.035, respectively), and a greater FEV_1_ decline occurred prior to graft loss (*p* < 0.0001). Overall, there was a median time to CLAD of 2.2 years (1.0–5.0) and median time to graft loss of 5.4 years (2.3–8.2), without significant difference between autopsy and redo-transplant patients (*p* = 0.34, *p* = 0.97, respectively). Histopathologic findings are reported for the combined group of autopsy and redo-lung transplant patients in BOS, a comparison of histologic findings between autopsy and redo-lung transplant patients is provided in Table 2. 

We also included explant lungs from 26 patients who died without CLAD, all collected at autopsy. The causes of death were cardiac (*n* = 8, 31%), pulmonary infection (*n* = 6, 23%), post-transplant lymphoproliferative disorder (*n* = 3, 12%), sepsis (*n* = 3, 12%), multi-organ-failure (*n* = 2, 8%), brain tumor (*n* = 2, 8%), subarachnoid hemorrhage (*n* = 1, 4%) and non-transplant related post-operative complication (*n* = 1, 4%). An overview of the patient characteristics and a comparison with BOS patients is provided in Table S2. In summary, non-CLAD patients were older compared to BOS patients (median: 58y (43–62) vs. 43y (27–57), *p* = 0.0025) and had a lower time to graft loss (median: 1.4 vs. 5.4 years, *p* = 0.0036). A complete overview of histological findings in non-CLAD lungs, and comparison with BOS explant lungs is provided in Table 2.

### 3.2. Airway-Centered Lesions

**Figure 2:**
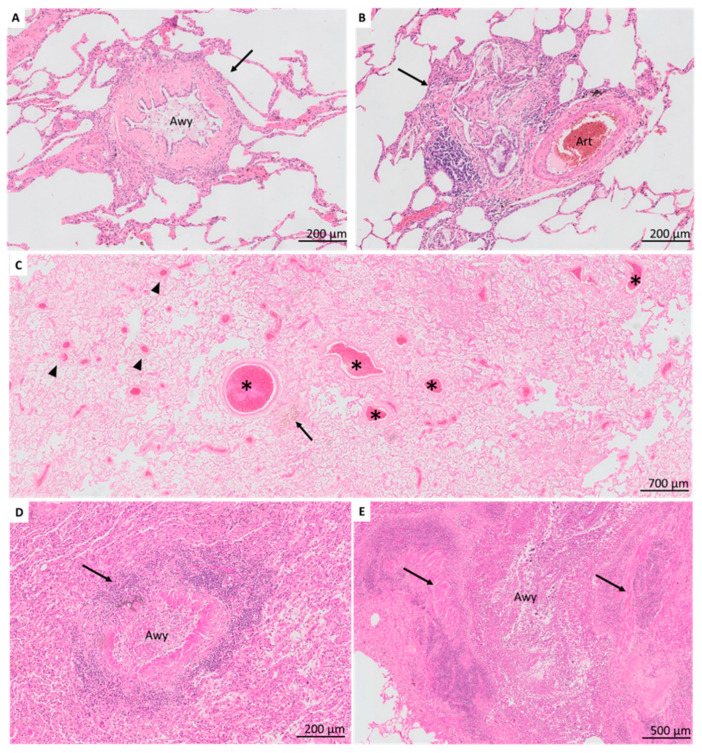
Airway lesions in BOS patients. (**A**). BO lesion with prominent concentric sub-epithelial fibrosis (arrow). The airway lumen is filled with foamy macrophages. (**B**). The airway is no longer recognizable but replaced by multiple prominent cholesterol clefts and marked inflammation (arrow). The accompanying artery is normally preserved and reveals the bronchocentric location of the cholesterol clefts. (**C**). Impression of vanishing airways. There are many recognizable larger vessels present (asterisk) without recognizable accompanying airways, and several small arteries and arterioles (arrowhead). A potential remnant of an airway is revealed by the presence of a collection of hemosiderin laden macrophages (arrow). (**D**). Prominent lymphocytic bronchiolitis, with a concentric presence of lymphocytes invading the airway wall (arrow). The airway lumen contains foamy macrophages. (**E**). Bronchiectasis recognized by a markedly distended airway lumen with extensive chronic inflammation diffusely present in the airway wall and lumen. The smooth muscle layer is still recognizable (arrows), surrounded by inflammatory infiltrates. Awy, airway; art, artery.

In BOS explant lungs, BO lesions were detected in 38 (73%) patients and were heterogeneous, ranging from BO with concentric sub-epithelial fibrosis (Figure 2A) to inflammatory BO, and cholesterol clefts distorting the airway lumen (Figure 2B). Vanishing airways (i.e., three arteries of >500 µm diameter without accompanying airway) were prominent in 13 (25%) patients and observed in 10/14 patients without detectable BO (Figure 2C). Small-airway lymphocytic bronchiolitis was present in 20 (38%) (B1R, *n* = 13; B2R, *n* = 7) (Figure 2D); large-airway lymphocytic bronchitis in 29 (56%) (E1, *n* = 13; E2, *n* = 16). Bronchiectasis was present in 10 (19%) patients (Figure 2E). Squamous metaplasia of the respiratory epithelium was noted in nine (17%) patients.

In non-CLAD patients, airway-centered lesions were limited, with detection of small airway lymphocytic bronchiolitis (B1R) and large airway bronchitis (E1) in only one (4%) patient, which was significantly less compared to BOS lungs (*p* = 0.00324 and *p* = 0.0038, respectively). Notably, no BO lesions, vanishing airways nor bronchiectasis were observed. Squamous metaplasia was present in three (12%) patients. 

### 3.3. Vascular Lesions

**Figure 3:**
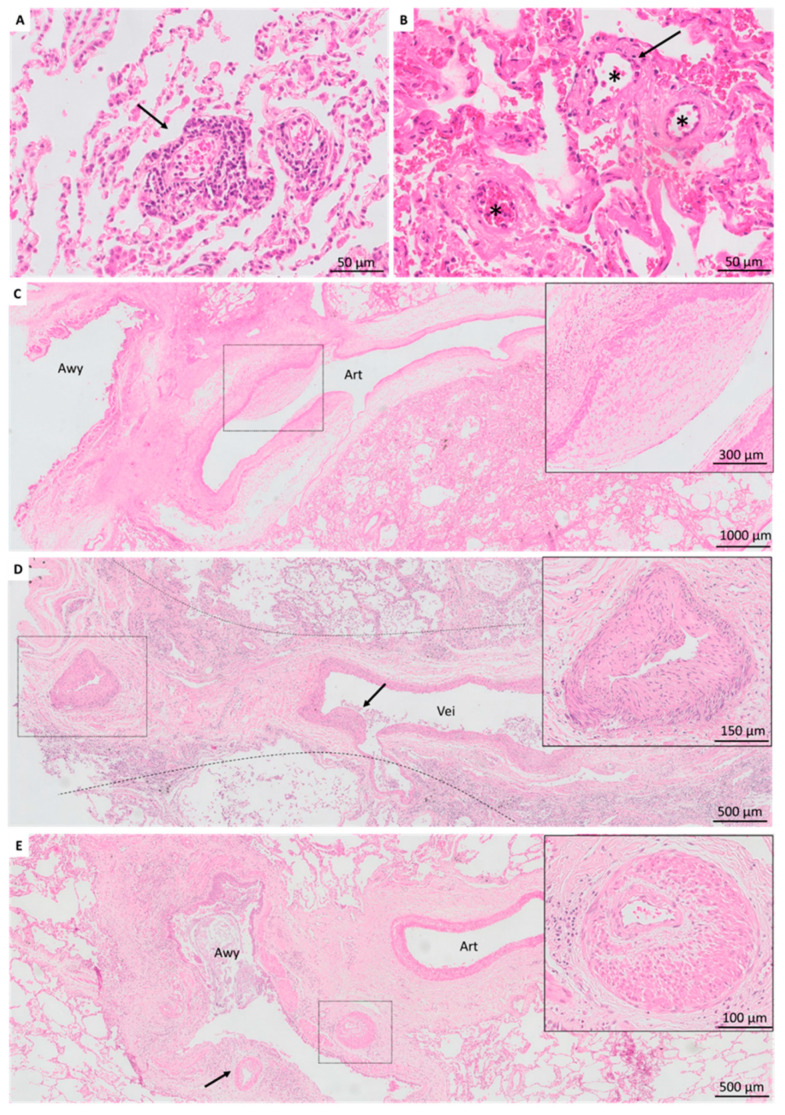
Vascular lesions in BOS patients. (**A**). Mild acute cellular rejection (grade A2), characterized by circumferential lymphocytic inflammation around small vessels. (**B**). Three small vessels (asterisk) with neutrophilic margination and suspicion of endothelitis, characterized by intimal thickening with few inflammatory cells in the subendothelial space (arrow) (**C**). Pulmonary arteriopathy, characterized by eccentric intimal fibrosis. (**D**). Venopathy, characterized by luminal obliteration of a smaller vein due to prominent concentric intimal proliferation, and eccentric intima proliferation in a larger vein. (**E**). Bronchial arterial vasculopathy with prominent media hypertrophy (arrow). Awy, airway; art, artery; vei, vein.

In BOS explant lungs, acute vascular lesions included detection of acute rejection in 8 (15%) patients (A1, *n* = 6; A2, *n* = 2) and microvascular injury (with endothelitis) in two (4%) patients (Figure 3A,B). Chronic vascular abnormalities were detected in 22 (42%) patients. More specifically, pulmonary arteriopathy was present in 17 (33%) patients, characterized by intimal fibrosis/hyperplasia, and additional media hypertrophy in 10 (19%) (Figure 3C). Pulmonary veins displayed venopathy, evidenced by intimal hyperplasia in 14 (27%), with venous occlusion in five (10%) patients (Figure 3D). Bronchial arteries displayed vasculopathy in 11 (21%) patients, all characterized by fibro-intimal hyperplasia and prominent media hypertrophy (Figure 3E).

In non-CLAD patients, acute vascular lesions were detected as acute rejection (A1) and microvascular injury in only one (4%) patient. Thrombi were present in five (19%) patients. Chronic vascular lesions were limited, with pulmonary arteriopathy evidenced by intima fibrosis without media hypertrophy in one (4%) patient, whereas pulmonary venopathy and bronchial vasculopathy were absent. 

### 3.4. Fibrotic Changes

**Figure 4:**
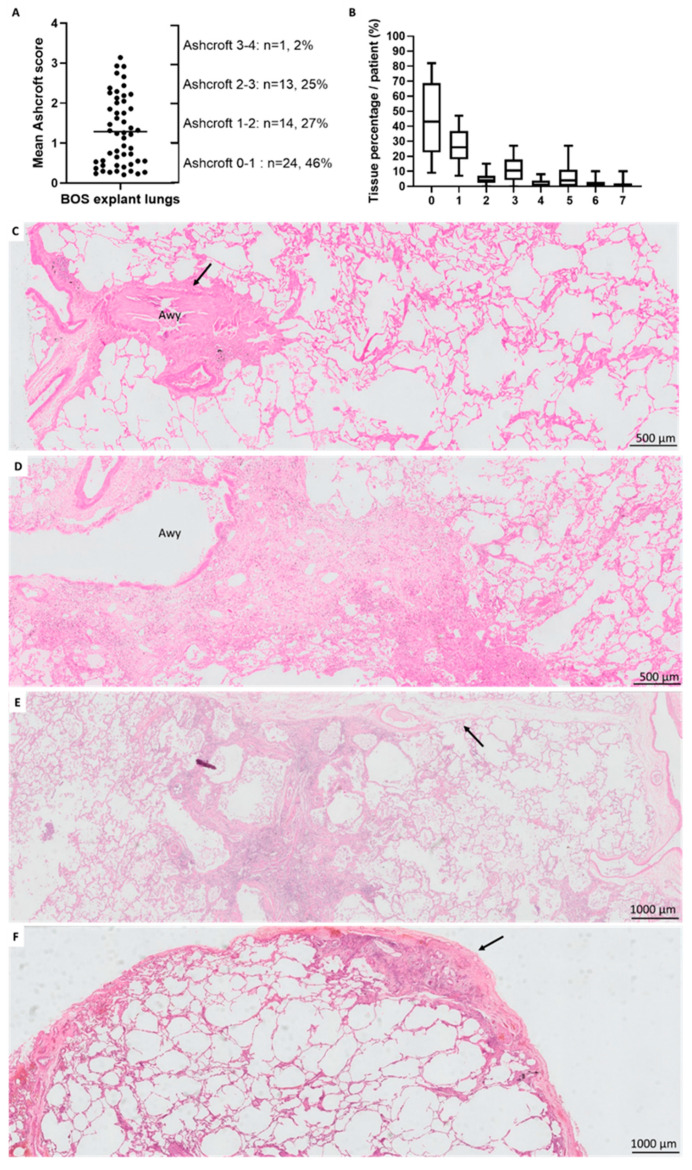
Fibrotic changes in BOS explant lungs. (**A**). Mean Ashcroft scores showed a large variation between patients, with an overall mean Ashcroft score of 1.29 (SD 0.87). (**B**). Distribution of the median tissue percentages of the different Ashcroft scores per patient, expressed as box plots displaying median, IQR and range. (**C**). BO lesion with typical sub-epithelial fibrosis (arrow), the surrounding parenchyma displays a partly mild thickening of the alveolar walls, without architectural damage. (**D**). Presence of prominent fibrosis organized around an airway, consistent with bronchocentric fibrosis. (**E**). Paraseptal fibrosis, a zone of prominent fibrotic changes with adjacent septum (arrow), with further adjacent inconspicuous lung parenchyma. (**F**). Subpleural fibrosis, with pleural thickening (arrow) and subpleural fibrosis, with surrounding emphysematous changes. Awy: airway.

Ashcroft fibrosis scoring was assessed on biopsies from three distinct locations in 50 (96%) BOS patients; for two (4%) BOS patients, only two distinct locations could be included. In BOS, a median number of 457 fields per patient were scored (IQR (408–496), range (268–601)). In non-CLAD lungs, three distinct locations were available for all patients, with a median of 464 analyzed fields per patient (IQR (422–513), range (342–610)). In BOS, Ashcroft score percentages showed a large variation between patients with an overall mean Ashcroft score of 1.29 (SD 0.87) (Figure 4A), without difference in mean Ashcroft scores between locations (*p* = 0.70). Analysis of non-CLAD lungs revealed an overall mean Ashcroft score of 0.48 (SD 0.49), without detection of location-specific differences (*p* = 0.95); which was significantly lower compared to BOS explant lungs (*p* = 0.0038, Figure S2).

In BOS, the median percentage of normal lung parenchyma (Ashcroft score 0) was 43% (IQR 23–69) per patient; minimal alveolar thickening (Ashcroft score 1) was 26% (IQR 18–37); and moderate alveolar thickening without definite architectural damage (Ashcroft 3) equaled 11% (IQR 4–18) (Figure 4B,C). Localized definite fibrotic damage of the lung structure (i.e., Ashcroft score ≥ 5 in ≥ 1 field) was present in 28 (54%) patients, and the general fibrosis pattern within this group was further (non-mutually exclusive) classified as bronchocentric fibrosis (*n* = 21/28, 75%), paraseptal fibrosis (*n* = 17/28, 61%) and subpleural fibrosis (*n* = 15/28, 54%) (Figure 4D–F, respectively). In these 28 patients with localized architectural damage, there was a median percentage of fibrosis (Ashcroft score ≥ 5) of 11% (IQR 8–17) and severe structural destruction with large fibrous areas (Ashcroft score 7) of 2% (IQR 0–4). In addition, localized alveolar fibrosis resembling non-specific interstitial pneumonia pattern was present in 2/52 (4%) patients (unrelated to recurrence of native lung disease as patients were transplanted for cystic fibrosis and emphysema). Fibroblast foci, evident honeycombing, usual interstitial pneumonia pattern or pleuroparenchymal fibro-elastosis were not observed. 

In non-CLAD patients, the median percentage of normal lung parenchyma (Ashcroft score 0) was 78% (IQR 64–88) per patient; minimal alveolar thickening (Ashcroft score 1) 15% (IQR 10–21); and moderate alveolar thickening without definite architectural damage (Ashcroft 3) 0% (IQR 0–3). Definite localized fibrotic damage to the lung structure was present in four (15%) patients, and non-mutually exclusive classified as paraseptal in two (8%) and subpleural in three (12%) patients, whereas bronchocentric fibrosis was not observed.

### 3.5. Other Histologic Findings

**Figure 5:**
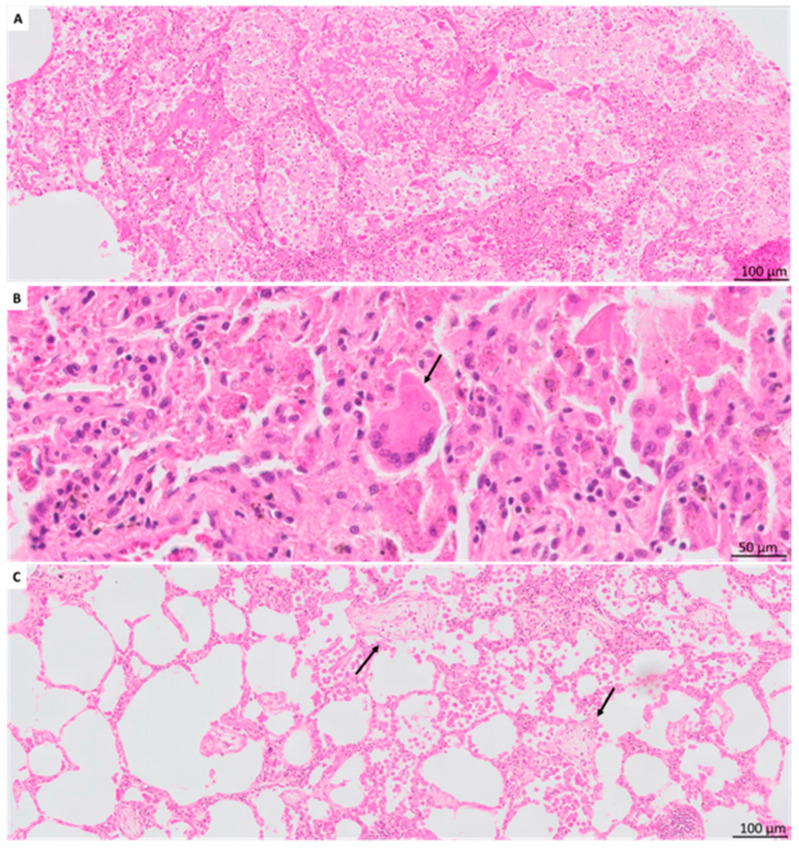
Other pathologic lesions in BOS explant lungs. (**A**). Evidence of prominent presence of intra-alveolar foamy macrophages, partly lipid laden. (**B**). Presence of an intra-alveolar giant cell (arrow). (**C**). Presence of organizing pneumonia characterized by several fibroblastic plugs (arrows). The surrounding alveolar walls display mild thickening.

Emphysema was significantly more frequent in BOS (*n* = 29, 56%) compared to non-CLAD (*n* = 4, 15%) (*p* = 0.027). Pneumocyte hyperplasia/reactive changes were present in 15 (29%) BOS and 9 (35%) non-CLAD patients. Intra-alveolar accumulation of foamy macrophages was observed in 10 (19%) BOS patients but absent in non-CLAD (Figure 5A). Giant cells were observed in 13 (25%) BOS and one (4%) non-CLAD patient (Figure 5B). Cholesterol clefts and cholesterol granulomas were present in eight (15%) and six (12%) BOS patients, respectively, but absent in non-CLAD. Intra-alveolar red blood cells were present in 27 (52%) BOS and 16 (62%) non-CLAD patients, with associated hemosiderin-laden macrophages in 15 (29%) BOS and three (12%) non-CLAD patients. Intra-alveolar neutrophils were more frequently observed in non-CLAD patients (*n* = 15, 58%) compared to BOS patients (*n* = 7, 13%) (*p* = 0.0038). Intra-alveolar fibrin deposition was identified in 13 (25%) BOS and 12 (46%) non-CLAD patients; organizing pneumonia in 11 (21%) BOS and 10 (38%) non-CLAD patients (Figure 5C); and acute fibrinous and organizing pneumonia in four (8%) BOS and one (4%) non-CLAD patient. In addition, hyaline membranes were present in five (10%) BOS and nine (35%) non-CLAD patients. Other histological findings are described in the Supplementary Materials. A comparison of histological findings in BOS explant lungs stratified per lung transplant era did not reveal any significant differences (Table S3).

## 4. Discussion

The current in-depth histopathologic characterization of explant lung tissue of well-defined clinical BOS patients and non-CLAD patients revealed (I) a broad spectrum of histologic lesions within well-defined clinical BOS patients, with predominant airway-centered lesions; (II) presence of prominent vasculopathy in BOS; (III) presence of a varying degree of fibrotic changes in BOS; whereas, (IV) non-CLAD patients displayed limited airway-centered lesions, vasculopathy and fibrotic changes.

To our knowledge, this study provides the largest in-depth histopathologic characterization of clinical BOS patients and the first to specifically exclude RAS and mixed phenotype patients. It may also form the first study comparing histopathologic changes in explant BOS lungs with non-CLAD patients. Furthermore, it forms the first study to histologically describe vanishing airways in BOS post lung transplantation, and the first to report the extent of a varying degree of fibrosis in clinical BOS patients. Our findings confirm and substantially extend previous findings and indicate that BOS should no longer be considered as a purely airway-centered disease. Instead, attention to the whole histologic spectrum is important, as concurrent vascular and interstitial changes may contribute to the observed lung allograft dysfunction and perhaps explain disease progression or interpatient variability in clinical presentation and evolution. Nevertheless, airway lesions form the main culprit for disease in BOS, and we found abundant BO in most patients. BO lesions varied from pure fibrotic lesions to inflammatory BO, as previously described by Martinu et al. [11]. The lower percentage of detected BO in patients with a lower BOS stage (i.e., autopsy cases), likely reflects the lower abundance of BO in these lungs and therefore the more early disease stage following BOS onset. Previously, our group described abundant BO lesions in end-stage BOS explant lungs with occlusion of approximately 50% of airways with a diameter between 1 and 2 mm, and even 73% of airways with a diameter of less than 1 mm [21]. However, the length of airway obstruction was reported to be highly variable; which was confirmed by Colombat et al. [22]. They demonstrated through histological 3D reconstruction that BO lesions only cause segmental obstruction of airways, therefore complicating histological diagnosis. Interestingly, not only abnormal airways were prominent in our cohort, but also an imbalance between the number of vessels and airways was observed. In the context of pulmonary graft-versus-host-disease after allogeneic hematopoietic stem cell transplantation, Meignin et al. recently published similar observations [23]. They described vanishing airways as they noted that in some patients, bronchioles appeared to have vanished, only revealing the location of a bronchiole by the adjacent pulmonary artery. We made an arbitrary restrictive definition of vanishing airways, although the relation of bronchovascular bundles might vary. Nevertheless, larger arteries (>500 µm) should be always accompanied by an airway. As smaller pulmonary arteries and arterioles are also generally accompanied by a bronchiole, and remnants of BO lesions were considered as present airways, our definition likely underestimates the true extent of vanishing airways. Moreover, this might also explain the low incidence of vanishing airways in redo-LTx patients, as the abundant presence of BO lesions complicated fulfillment of the preset criteria. The observation of ‘vanishing airways’ might be accounted for by bronchiolar collapse lesions as previously identified on microCT [21]. These typically show only discrete scar-like luminal occlusions of airways without BO fibroproliferative tissue, and are not identifiable on routine histology [21]. Vanishing airways may also add to the low diagnostic potential of transbronchial biopsy sampling to identify BO lesions [24]. Of interest, no BO lesions or vanishing airways were detected in non-CLAD patients, which supports the proposed relation between airway lesions and FEV1 decline, and therefore the use of pulmonary function testing to diagnose CLAD. 

Chronic vascular rejection is generally considered as accelerated graft vascular sclerosis and termed grade-D rejection since the 1996 ISHLT criteria [18,25]. However, chronic vascular rejection cannot be assessed on transbronchial biopsies as it requires larger (surgical) biopsies. Therefore, only limited data is available on the vasculopathy of larger vessels. In 2008, Saggar and colleagues reported clinical pulmonary hypertension in 72% of transplant patients with pathologic BO lesions, and reported histologic arterial and venous abnormalities of the larger vessels in approximately 80% of patients with concurrent BO lesions [12]. The arteriopathy predominantly consisted of abnormalities of the intimal layer, with nonlaminar eccentric and concentric intimal proliferation and fibrosis; and to a lesser extent the medial layer. Venopathy consisted of diffuse intimal fibrosis with (sub)total luminal obliteration, perivenous lymphocytic infiltration and secondary capillary congestion. Our findings confirm the presence of prominent pulmonary arteriopathy and venopathy, although to a lesser extent, in respectively 33% and 27% of BOS patients. This difference is likely partly explained by specific exclusion of RAS in our cohort. In comparison, in non-CLAD patients, chronic vascular lesions were only present in a single patient. However, this difference was not significant after conservative post-hoc testing, likely due to a relative low sample size. Interestingly, we also found evidence of severe bronchial vasculopathy in 21% of BOS patients, characterized by fibro-intimal thickening and media hypertrophy, which was absent in non-CAD patients, although this difference was also not significant. Outside the context of lung transplantation, bronchial arteries have been reported to be resistant to atherosclerosis, presumably due to abundant collateral circulation [26]. However, as bronchial arterial re-anastomosis was not performed, bronchial arteries were dependent on collateral circulation from the pulmonary circulation. The significance of this finding remains unclear, but it might potentially serve as an indirect sign of airway hypoxia in larger airways. Overall, our findings support increased attention for the role of the vascular component in BOS.

A striking observation was the presence of a varying degree of fibrosis in our BOS patients. There was large variation between patients, but all displayed at least minimal fibrotic interstitial thickening. In approximately half of the patients, microscopic fibrotic foci distorted the lung structure. Although the nature and potential evolution of these lesions remains unknown, it might support the hypothesis of a potential continuum between BOS and RAS. Recent consensus guidelines indeed describe both an undefined and mixed phenotype of CLAD, and a continuum between BOS and RAS is speculated [3]. The actual transition from BOS to the mixed phenotype remains poorly understood, but one could speculate that progression of fibrotic lesions might lead to persistent radiological opacities and ultimately even restrictive pulmonary function deficit. However, pleuroparenchymal fibro-elastosis, the dominant fibrotic pattern in RAS, was not observed in BOS [14,27]. 

This study illustrates the presence of both vascular and fibrotic changes in an important subset of BOS patients. While the nature and evolution of these lesions remains to be addressed and falls beyond the scope of the current manuscript, the relation between fibrotic lung remodeling and vasculopathy is of particular interest for further research. This complex pathophysiological interaction has been a topic of increasing interest in idiopathic pulmonary fibrosis. While the pathophysiology largely remains to be elucidated, in part due to the complex temporal and geographical distribution of vascular and fibrotic lesions, several cellular and molecular mechanisms are likely involved [27]. Fibrotic lung remodeling itself may cause loss of microvasculature and chronic hypoxia, resulting in chronic hypoxic vasoconstriction, over time resulting in increased muscle wall thickness and pulmonary hypertensive changes [28]. Interestingly, fibrosis and pulmonary arterial vasculopathy share many pathogenic mechanisms and progression can be induced by the same mediators (e.g., vascular endothelial growth factor, transforming growth factor beta, nitric oxide) [29,30]. Alternatively, vasculopathy may proceed fibrotic changes. With regard to CLAD, ultrastructural evidence of chronic septal vasculopathic changes was indeed found to be an important predictor of chronic graft dysfunction [31,32]. Endothelial-to-mesenchymal transition (EMT) forms another potential pathophysiologic mechanism linking vasculopathy and fibrosis. EMT refers to a state in which the endothelial cells lose their endothelial phenotype in response to an external stimulus or internal pathologic condition [33], and progress to a mesenchymal state with creation of a profibrotic environment with accumulation of collagen-producing myofibroblasts [27]. As lung transplantation forms a specific clinical setting, in which there is a global pro-coagulative state with chronic endothelial activation [34], this may, at least theoretically, predispose to vasculopathy. It may be evident that more research is needed on unravelling the complex interplay between pulmonary fibrosis and vasculopathy.

Our study has some limitations. First, patients from a single center and tissue collected over a long time-period were included. Despite the large cohort, extrapolation of results might therefore be limited. In addition, therapeutic and surgical management changed over time which may affect BOS onset or progression (e.g., azithromycin prophylaxis), although histologic findings did not significantly differ between different transplant eras. Secondly, only patients with graft loss could be included and results might not be representative for the whole spectrum of BOS patients. Third, non-CLAD patients had a significant lower graft survival compared to BOS patients due to inherent limitations in collecting explant lung tissue. This needs to be taken into account when comparing histological findings, and it is likely that certain non-significant histologic differences might become significant in a future larger cohort analysis. Furthermore, fibrotic changes may present as heterogeneous. While we performed an elaborate Ashcroft fibrosis scoring technique, which did not reveal location-specific differences, potential heterogeneity must be taken into account. Finally, patients experienced potential acute events before graft loss, which may be reflected in acute histologic findings.

In conclusion, the present study reports an in-depth histopathologic characterization of clinically well-defined BOS patients and describes various histopathologic findings, including airway lesions, vasculopathy and fibrotic changes. Exploration of histopathologic findings beyond BO might provide novel insight in the pathophysiology of BOS, the observed clinical interpatient variability in BOS and its potential histopathologic relationship with RAS. 

## Figures and Tables

**Table 1:** Overview of patient characteristics of BOS patients.

	Total	Autopsy	Redo-LTx	*p* Value
**Patients, *N***	52	23	29	
**Age at transplant (y)**	43 (27–57)	56 (39–60)	30 (25–47)	**0.0077**
**Male, *N* (%)**	21 (40)	7 (30)	14 (48)	0.43
**Underlying disease, *N* (%)**				
Emphysema	17 (33)	14 (61)	3 (10)	**0.0002**
ILD	10 (19)	4 (17)	6 (21)	>0.99
CF + BRECT	19 (37)	2 (9)	17 (59)	**0.0003**
PHT + Eisenmenger	4 (8)	3 (13)	1 (3)	0.31
Other	2 (4)	0	2 (7)	0.50
**Type of transplant, *N* (%)**				
BLTx	37 (71)	11 (48)	26 (90)	**0.016**
HLTx	4 (8)	3 (13)	1 (3)	0.31
SLTx	11 (21)	9 (39)	2 (7)	**0.0066**
**Time to CLAD (y)**	2.2 (1.0–5.0)	3.5 (1.1–6.0)	1.5 (1.0–3.7)	0.34
**Time to graft loss (y)**	5.4 (2.3–8.2)	5.5 (2.4–8.6)	4.8 (2.2–7.7)	0.97
**Graft survival after CLAD onset (y)**	1.4 (0.6–3.7)	1.1 (0.3–2.3)	2.1 (0.7–4.4)	0.29
Time BOS1 to BOS3 (y) *	0.4 (0.1–1.5)	0.8 (0.2–1.7)	0.3 (0.1–1.4)	0.43
**Time BOS3 to graft loss (y)**	0.9 (0.5–2.3)	0.6 (0.1–2.2)	1.1 (0.5–2.5)	0.23
**Post-LTx best FEV_1_ (L)**	2.4 (1.9–3.1)	2.1 (1.5–2.8)	2.8 (2.3–3.6)	**0.0012**
**Post-LTx best FVC (L)**	3.3 (2.6–4.0)	2.9 (2.4–3.8)	3.4 (2.9–4.2)	**0.035**
**FEV_1_ before graft loss (L)**	0.6 (0.5–0.9)	0.8 (0.6–1.3)	0.6 (0.5–0.8)	0.12
**FEV_1_ decline (%)**	72.1 (60.8–79.8)	60.8 (40.3–67.2)	77.4 (72.8–80.8)	**<0.0001**
**Time last FEV1 to graft loss (d)**	29 (10–47)	44 (24–59)	18 (8–39)	**0.015**
**CLAD (BOS) stage before graft loss**				
Stage 1	5 (10)	4 (17)	1 (3)	0.16
Stage 2	4 (8)	4 (17)	0	**0.033**
Stage 3	6 (12)	6 (26)	0	**0.0050**
Stage 4	37 (71)	9 (39)	28 (97)	**<0.0001**
**Ever AR, *N*(%)**	30 (58)	12 (52)	18 (62)	0.58
Ever severe AR (≥A2), *N*(%)	16 (31)	6 (26)	10 (34)	0.56
**Ever LB, *N*(%)**	17 (33)	7 (30)	10 (34)	>0.99
Ever severe LB (=B2R), *N*(%)	9 (17)	4 (17)	5 (17)	>0.99
**History of DSAs, *N*(%)**	6 (12)	3 (13)	3 (10)	>0.99
BAL at BOS1 diagnosis **				
Total cell count (10³/mL)	151.0 (69.0–280.0)	151.0 (80.0–697.5)	155.5 ( 55.25–247.0)	0.77
Macrophages %	70.4 (49.2–90.6)	61.8 (34.3–90.8)	75.7 (54.9–89.6)	0.80
Lymphocytes %	6.0 (2.2–12.4)	5.5 (0.9–18.4)	6.3 (3.2–11.4)	0.88
Neutrophils %	8.0 (3.0 -39.0)	18.0 (2.5–60.2)	7.2 (3.4–35.7)	0.89
Eosinophils %	0.2 (0.0–1.0)	0.0 (0.0–0.4)	0.2 (0.0–2.0)	0.16

Data are shown as *n*, *n* (%), and median (interquartile range). Y: years; d: days; LTx: lung transplantation; ILD: interstitial lung disease; CF: cystic fibrosis; BRECT: bronchiectasis; PHT: pulmonary hypertension; BLTx: bilateral lung transplantation; HLTx: heart-lung transplantation; SLTx: single-lung transplantation; CLAD: chronic lung allograft dysfunction; BOS: bronchiolitis obliterans syndrome; AR: acute rejection; LB: lymphocytic bronchiolitis; DSA: donor-specific antibodies; BAL: broncho-alveolar lavage. (*) Three patients were immediately diagnosed with BOS stage 3, without prior BOS stage 1 diagnosis. Nine patients did not develop BOS stage 3 before graft loss. (**) BAL at BOS stage 1 diagnosis was not available for nine patients.

**Table 2:** Comparison of histological findings in BOS vs. non-CLAD explant lungs. Data are shown as n, n (%), and mean (standard deviation). Histological findings were compared using the Fisher Exact test and Mann Whitney test, with Bonferroni-Dunn post-hoc testing.

	BOS: Autopsy vs. Redo-LTx			BOS vs. Non-CLAD		
	BOS Autopsy	BOS Redo-LTx	Adjusted *p* Value	BOS Total	Non-CLAD	Adjusted *p* Value
**Patients, *N***	23	29		52	26	
**Airway lesions**						
Small-airway lymphocytic bronchiolitis	3 (13)	17 (59)	0.051	20 (38)	1 (4)	**0.034**
Large-airway lymphocytic bronchitis	13 (57)	16 (55)	1.00	29 (56)	1 (4)	**0.0038**
Bronchiolitis obliterans	9 (39)	29 (100)	**0.0039**	38 (73)	0	**0.0038**
Vanishing airways	12 (52)	1 (3)	**0.0039**	13 (25)	0	0.13
Bronchiectasis	1 (4)	9 (31)	1.00	10 (19)	0	0.98
Follicular bronchiolitis	0	1 (3)	1.00	1 (2)	0	1.00
Mucus plugs	6 (26)	13 (45)	1.00	19 (37)	16 (62)	1.00
Squamous metaplasia	4 (17)	5 (17)	1.00	9 (17)	3 (12)	1.00
Respiratory bronchiolitis	0	2 (7)	1.00	2 (4)	1 (4)	1.00
**Vascular lesions**						
Acute rejection	6 (26)	2 (7)	1.00	8 (15)	1 (4)	1.00
Pulmonary arteriopathy	7 (30)	10 (34)	1.00	17 (33)	1 (4)	0.15
*Media hypertrophy*	2 (9)	8 (28)	1.00	10 (19)	0	0.98
*Intima hyperplasia*	7 (30)	10 (34)	1.00	17 (33)	1 (4)	
Pulmonary venopathy	4 (17)	10 (34)	1.00	14 (27)	0	0.13
*Intima hyperplasia*	4 (17)	10 (34)	1.00	14 (27)	0	0.13
*Venous occlusion*	2 (9)	3 (10)	1.00	5 (10)	0	1.00
Bronchial arteriopathy	2 (9)	9 (31)	1.00	11 (21)	0	0.49
*Media hypertrophy*	2 (9)	9 (31)	1.00	11 (21)	0	0.49
*Intima hyperplasia*	2 (9)	9 (31)	1.00	11 (21)	0	0.49
Microvascular injury	0	2 (7)	1.00	2 (4)	1 (4)	1.00
Thrombi	1 (4)	2 (7)	1.00	3 (6)	5 (19)	1.00
**Alveolar compartment**						
Pneumocyte hyperplasia	3 (13)	12 (41)	1.00	15 (29)	9 (35)	1.00
Emphysema	11 (48)	18 (62)	1.00	29 (56)	4 (15)	**0.027**
Hemosiderophages	6 (26)	9 (31)	1.00	15 (29)	3 (12)	1.00
Neutrophils in alveoli	5 (22)	2 (7)	1.00	7 (13)	15 (58)	**0.0038**
Eosinophils in alveoli	0	1 (3)	1.00	1 (2)	0	1.00
Organizing pneumonia	6 (26)	5 (17)	1.00	11 (21)	10 (38)	1.00
AFOP	3 (13)	1 (3)	1.00	4 (8)	1 (4)	1.00
Hyaline membranes	5 (22)	0	0.55	5 (10)	9 (35)	0.18
Cholesterol clefts	2 (9)	6 (21)	1.00	8 (15)	0	1.00
Giant cells	2 (9)	11 (38)	0.90	13 (25)	1 (4)	0.94
RBCs intra-alveolar	13 (57)	14 (48)	1.00	27 (52)	16 (62)	1.00
Fibrin intra-alveolar	9 (39)	4 (14)	1.00	13 (25)	12 (46)	1.00
Foamy macrophages intra-alveolar	1 (4)	9 (31)	1.00	10 (19)	0	0.98
**Fibrotic lesions**						
Mean Ashcroft score	1.26 (SD 1.04)	1.306 (SD 0.72)	1.00	1.29 (SD 0.87)	0.48 (SD 0.49)	**0.0038**
Ashcroft score ≥5	10 (43)	18 (62)	1.00	28 (54)	4 (15)	0.053
*Bronchocentric fibrosis*	5 (22)	16 (55)	0.090	21 (40)	0	**0.0038**
*Paraseptal fibrosis*	8 (35)	9 (31)	1.00	17 (33)	2 (8)	0.89
*Subpleural fibrosis*	6 (26)	9 (31)	1.00	15 (29)	3 (12)	1.00

## Data Availability

The data that support the findings of this study are available on request from the corresponding author.

## Supplementary Materials

The following are available online at https://www.mdpi.com/article/10.3390/jcm11010111/s1, Figure S1: Ashcroft scoring method for BOS explant lungs; Figure S2: Comparison of overall Mean Ashcroft scores between non-CLAD and BOS explant lungs; Table S1: Histological scoring grid for BOS explant lungs and applied histological definitions; Table S2: Comparison of patient characteristics between BOS and non-CLAD patients; Table S3: Histological findings in BOS lungs stratified per transplant era.
