# Supplementary material for: Beyond Bronchiolitis Obliterans: In-Depth Histopathologic Characterization of Bronchiolitis Obliterans Syndrome after Lung Transplantation
Source: J Clin Med. 2021 Dec 27;11(1):111. doi: 10.3390/jcm11010111 (PMC8745215; doi:10.3390/jcm11010111)
Supplement: Supplementary file 1 [file jcm-11-00111-s001.zip › jcm-1503384-supplementary.pdf]

Supplementary

"BEYOND BRONCHIOLITIS OBLITERANS: IN-DEPTH HISTOPATHOLOGIC CHARACTERIZATION OF BRONCHIOLITIS OBLITERANS SYNDROME AFTER LUNG TRANSPLANTATION"

Vanstapel A, Verleden SE, et al.

Methods

Ashcroft scoring of fibrosis

Fibrotic changes were quantified by assessment of the Ashcroft score according to the original scoring system by Ashcroft et al. in 1988, with minimal modifications (i.e. bronchiolar wall thickening was not assessed, no distinction was made between Ashcroft score 7 and 8, and intra-alveolar organized exudates were not considered as fibrosis)[4]. For each 2 mm² field, the predominant Ashcroft score was noted (no score was noted when the field was predominantly occupied by large bronchi or vessels). Ashcroft score 0 refers to normal preserved lung parenchyma. Ashcroft score 1 indicates minimal fibrous thickening of the alveolar walls. Fibrous thickening of bronchiolar walls was originally also considered as Ashcroft score 1 by Ashcroft et al, but was not included in our analysis as we were interested in alveolar fibrotic remodeling in BOS patients, and bronchiolar wall assessment (especially BO lesions) would influence these results. Ashcroft score 2 is noted when there is difficulty deciding between Ashcroft 1 and 3. Aschroft score 3 refers to moderate thickening of alveolar walls without obvious damage to lung structure (bronchiolar wall thickening was not included). Ashcroft score 4 refers to difficulty deciding between Ashcroft 3 and 5. Ashcroft score 5 refers to fibrosis with definite damage to the lung structure and formation of fibrous bands/small fibrous masses. Ashcroft score 6 refers to difficulty deciding between Ashcroft 5 and 7. Ashcroft score 7 referred to severe distortion of the lung structure and presence of large fibrous areas (including honeycombing). We did not make an additional distinction between Ashcroft score

7 and Ashcroft score 8 (total fibrous obliteration of the field) and placed these both under Ashcroft score 7. The mean Ashcroft score was determined using the following formula: mean Ashcroft score = ((1 x Ashcroft score 1 tissue %) + (2 x Ashcroft score 2 tissue %) + (3 x Ashcroft score 3 tissue %) + (4 x Ashcroft score 4 tissue %) + (5 x Ashcroft score 5 tissue %) + (6 x Ashcroft score 6 tissue %) + (7 x Ashcroft score 7 tissue %))/100.

Results

Autopsy rates

In total, graft loss occurred in 476 of 1092 (44%) patients. Of the 476 patients with graft loss, 54 (11%) were redo lung transplant patients and 422 (89%) died. An autopsy procedure was performed in 139 of 422 (33%) deceased patients.

Other histologic findings

Histologic evidence of infectious organisms were identified in a subset of both BOS and non-CLAD patients. Pulmonary aspergillosis was identified in 3 (6%) BOS and 6 (23%) non-CLAD patients, cytomegalovirus inclusions in 2 (4%) BOS and 5 (19%) non-CLAD patients. Further, in the BOS cohort, we found pneumocystis carinii pneumonia in one (2%) patient, necrotizing granulomas due to atypical mycobacterial infection in one (2%) patient, and angio-invasive mucormycosis in one (2%) patient. Of the 6 BOS patients that presented with pulmonary infection, there was concomitant presence of hyaline membranes in 1/6, organizing pneumonia in 1/6, intra-alveolar fibrin in 2/6 and intra-alveolar foamy macrophages in 2/6 that showed (partial) overlap with zones of infection. Other miscellaneous findings in BOS included presence of prominent bone marrow emboli in one (2%) patient with prior cardiopulmonary resuscitation. Blue bodies, asteroid bodies and osseous metaplasia were each observed in one (2%) BOS patient. A tumorlet was present in 1 (4%) non-CLAD patient.

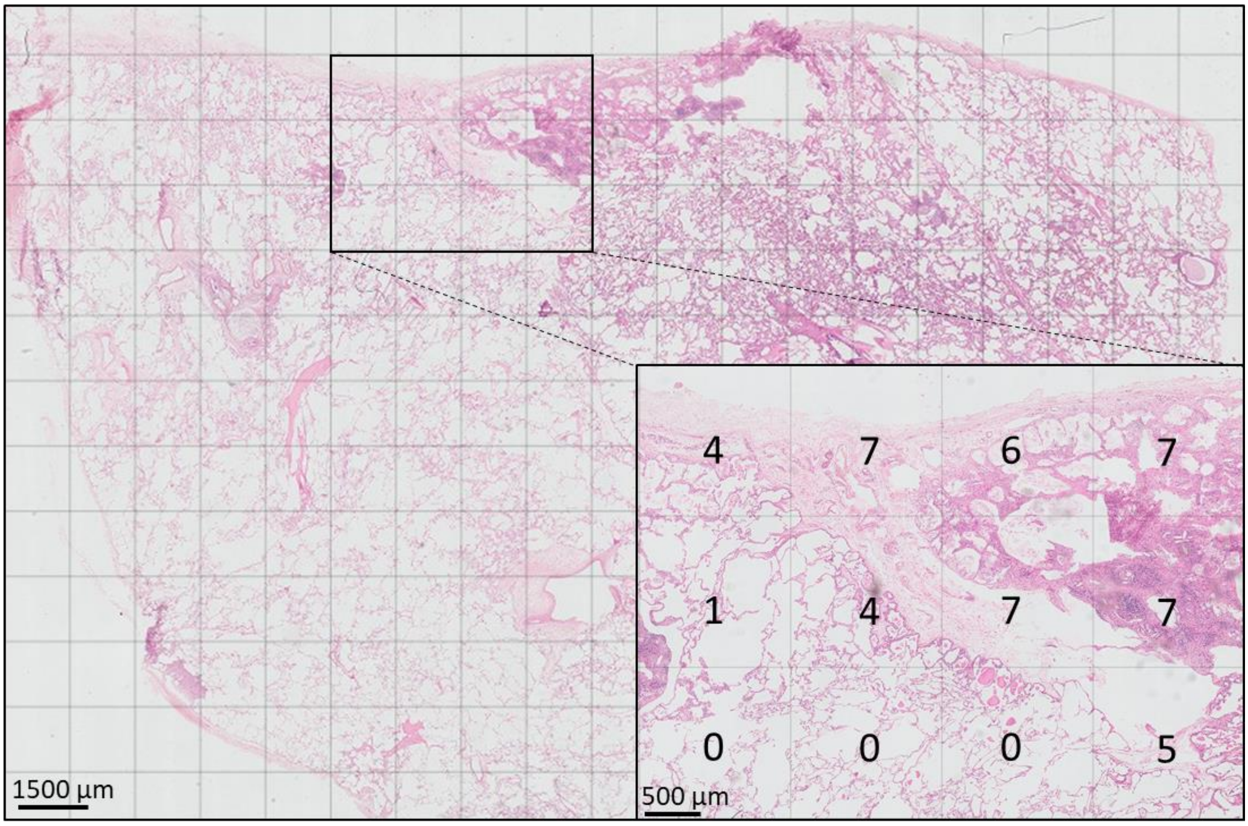

Figure S1. Ashcroft scoring method for BOS explant lungs. A scoring grid was digitally projected on the scanned slides and the Ashcroft score was determined for each 2 mm² field of the entire tissue section. The inset highlights a zone with partial prominent fibrotic changes, with the Ashcroft scores noted for each individual field. As an Ashcroft score of ≥5 was present, the fibrotic pattern was additionally categorized as subpleural fibrosis in this explant lung.

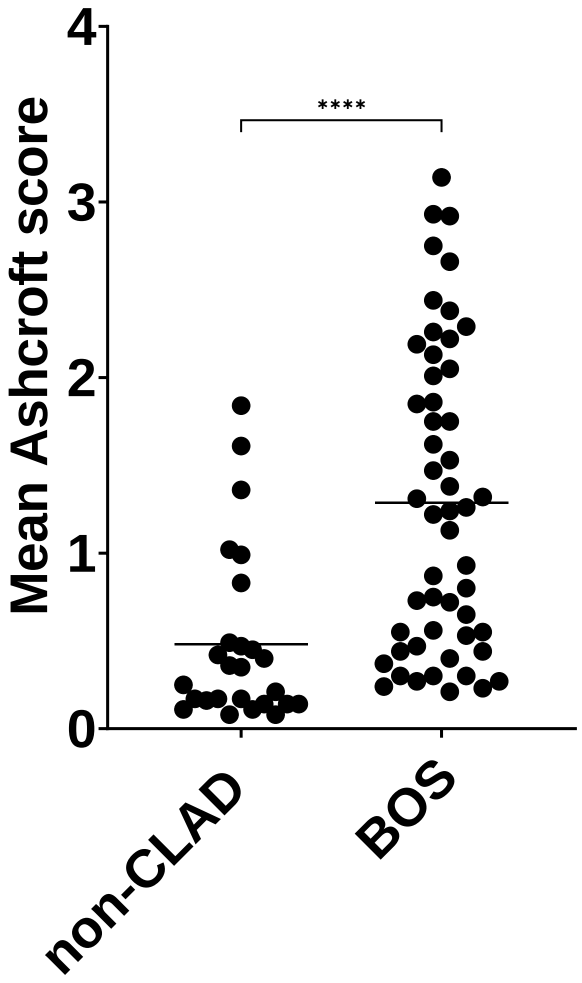

Figure S2. Comparison of overall Mean Ashcroft scores between non-CLAD and BOS explant lungs. There was a significant increase in the mean Ashcroft scores in BOS explant lungs (overall mean: 1.29, SD: 0.87) compared to non-CLAD explant lungs (overall mean: 0.48, SD: 0.49) (univariate $p<0.0001$, after Bonferroni-Dunn post-hoc testing $p=0.0038$).

**Table S1.** Histological scoring grid for BOS explant lungs and applied histological definitions			
**Airway lesions**			
Lymphocytic bronchiolitis (B grade)<br>*Small airway inflammation graded as B0, B1R, B2R, based on the 2007 ISHLT working formulation for diagnosing lung rejection*[1]		Follicular bronchiolitis<br>*Prominent peribronchial lymphocytic infiltration with formation of multiple tertiary lymphoid follicles*[5]	
Large-airway bronchiitis (E grade)		Mucus plugs<br>*Intrabronchiolar presence of thickened mucus, with/without accompanying cell debris*	
Bronchiolitis obliterans<br>*Fibrosing process involving the small bronchioles causing sub-epithelial fibrotic proliferation and (sub)total obliteration with or without accompanying inflammation*[3]		Squamous metaplasia<br>*(partial) Replacement of normal respiratory epithelium by stratified squamous epithelium*[6]	
Vanishing airways<br>*≥3 larger arteries (vessel diameter > 500 µm) without accompanying airway (BO lesions were counted as presence of an airway)*		Respiratory bronchiolitis<br>*Yellow-brown pigmented macrophages around distal airways and peribronchiolar airspaces*[7]	
Bronchiectasis<br>*Abnormal bronchial dilatation with chronic inflammation and bronchial wall destruction (including muscular and elastic tissue)*[4]		Other airway lesions	
**Vascular compartment**			
AR (A grade)<br>*Acute cellular rejection graded as A0-A4) based on the 2007 ISHLT working formulation for diagnosing lung rejection*[1]		Microvascular injury (endothelitis)<br>*Neutrophilic capillaritis and endothelitis, not limited to neutrophilic margination and congestion*[1, 10]	
Pulmonary arteriopathy<br>*Media hypertrophy*<br>*Increased medial thickening by smooth muscle hypertrophy and hyperplasia, with/without matrix deposition*[8,9]<br>*Intima hyperplasia*<br>*Eccentric or concentric intimal hyperplasia or fibrosis*[1,8,9]		Pulmonary venopathy<br>*Intima hyperplasia*<br>*Eccentric or concentric intimal hyperplasia or fibrosis, usually visible as poorly cellular hyaline sclerosis*[1,8,9]<br>*Venous occlusion*<br>*Severe eccentric or concentric intimal hyperplasia or fibrosis leading to luminal occlusion*[9]	

Bronchial arteriopathy<br><br>*Media hypertrophy*<br><br>*Increased medial thickening by smooth muscle hypertrophy and hyperplasia, with/without matrix deposition[8,9]*<br><br>*Intima hyperplasia*<br><br>*Eccentric or concentric intimal hyperplasia or fibrosis[1, 8, 9]*		Thrombi<br><br>Other vascular findings	
**Alveolar compartment**			
Pneumocyte hyperplasia/reactive changes<br><br>*Enlarged type II pneumocytes with eosinophilic cytoplasm, nuclear enlargement and potential distinct nucleoli[11]*		Hyaline membranes<br><br>*Dense amorphous eosinophilic membranes which closely line the alveolar walls[17]*	
Emphysema<br><br>*Enlargement of alveolar spaces and fragmentation of alveolar walls[12]*		Cholesterol clefts<br><br>*Characteristic needle-like spicular open spaces*	
Hemosiderophages<br><br>*Hemosiderin (golden-brown coarse pigment) laden macrophages[13]*		Cholesterol granuloma<br><br>*Multiple cholesterol clefts with with multinucleated giant cells and epithelioid histocytes*	
Neutrophils in alveoli		Giant cells<br><br>*Multinucleated epithelioid histiocytes*	
Eosinophils in alveoli		RBCs in alveolar spaces	
Fibroblast foci<br><br>*Linear arranged myofibroblast proliferation with characteristic pale gray matrix, usually in direct proximity of more advanced fibrosis[14]*		Fibrin in alveolar spaces	
OP<br><br>*Loose fibroblastic plugs within alveoli or alveolar ducts[15]*		Intra-alveolar foamy macrophages<br><br>*Lipid-laden macrophages with foamy cytoplasm*	
AFOP		Other findings	

Dominant finding of organizing intra-alveolar fibrin and organizing pneumonia, in a patchy distribution with only mild interstitial inflammation[16]			
**Assessment of fibrosis**			
N of assessed slides		N of assessed 2 mm² fields	
Ashcroft score 0 (N fields)<br>*Normal preserved lung parenchyma*[18]		Ashcroft score 4 (N fields)<br>*Difficulty deciding between Ashcroft 3 and 5*[18]	
Ashcroft score 1 (N fields)<br>*Minimal fibrous thickening of the alveolar walls*[18]		Ashcroft score 5 (N fields)<br>*Fibrosis with definite damage to the lung structure and formation of fibrous bands/small fibrous masses*[18]	
Ashcroft score 2 (N fields)<br>*Difficulty deciding between Ashcroft 1 and 3*[18]		Ashcroft score 6 (N fields)<br>*Difficulty deciding between Ashcroft 5 and 7*[18]	
Ashcroft score 3 (N fields)<br>*Moderate thickening of alveolar walls without obvious damage to lung structure (bronchiolar wall thickening was not included)*[18]		Ashcroft score 7 (N fields)<br>*Severe distortion of the lung structure and presence of large fibrous areas (including honeycombing)*[18]	
If Ashcroft score ≥5, define location of fibrosis (non-mutually exclusive)<br>*Bronchocentric fibrosis*<br>*Paraseptal fibrosis*<br>*Subpleural fibrosis*		Other fibrotic patterns<br>*PPFE*<br>*Hypocellular collagen deposition with a preserved alveolar septal elastic network*[19]<br>*UIP*<br>*Marked fibrosis and architectural distortion (including honeycomb changes) with a patchy and temporal heterogenous involvement*[20]<br>*NSIP*<br>*Temporal homogenous and diffuse interstitial fibrosis*[21]<br>*Other*	
**Other**			
Ischemic necrosis		Viral inclusions, type	

Fungal organisms, type		Other infectious organisms, type	
Aspirated foreign material		Granuloma<br>○ Necrotizing<br>○ Non-necrotizing<br>○ Isolated/multiple<br>○ Etiology	
Other observations			

References on which applied histological definitions were based:

1. Stewart S, Fishbein MC, Snell GI, Berry GJ, Boehler A, Burke MM, Glanville A, Gould FK, Magro C, Marboe CC, McNeil KD, Reed EF, Reinsmoen NL, Scott JP, Studer SM, Tazelaar HD, Wallwork JL, Westall G, Zamora MR, Zeevi A, Yousem SA. Revision of the 1996 working formulation for the standardization of nomenclature in the diagnosis of lung rejection. J Heart Lung Transplant. 2007 Dec;26(12):1229-42. doi: 10.1016/j.healun.2007.10.017. PMID: 18096473.
2. Greenland JR, Jones KD, Hays SR, et al. Association of large-airway lymphocytic bronchitis with bronchiolitis obliterans syndrome. Am J Respir Crit Care Med. 2013;187(4):417-423. doi:10.1164/rccm.201206-1025OC
3. Epler GR, Colby TV. The spectrum of bronchiolitis obliterans. Chest. 1983 Feb;83(2):161-2. doi: 10.1378/chest.83.2.161. PMID: 6822090.
4. Kang EY, Miller RR, Müller NL. Bronchiectasis: comparison of preoperative thin-section CT and pathologic findings in resected specimens. Radiology. 1995 Jun;195(3):649-54. doi: 10.1148/radiology.195.3.7753989. PMID: 7753989.
5. Vos R, Vanaudenaerde BM, De Vleeschauwer SI, Van Raemdonck DE, Dupont LJ, Verbeken EK, De Wever W, Verleden GM. Follicular bronchiolitis: a rare cause of bronchiolitis obliterans syndrome after lung transplantation: a case report. Am J Transplant. 2009 Mar;9(3):644-50. doi: 10.1111/j.1600-6143.2008.02518.x. Epub 2009 Feb 3. PMID: 19191770.
6. Rigden HM, Alias A, Havelock T, et al. Squamous Metaplasia Is Increased in the Bronchial Epithelium of Smokers with Chronic Obstructive Pulmonary Disease. PLoS One. 2016;11(5):e0156009. Published 2016 May 26. doi:10.1371/journal.pone.0156009
7. Niewoehner DE, Kleinerman J, Rice DB. Pathologic changes in the peripheral airways of young cigarette smokers. N Engl J Med. 1974 Oct 10;291(15):755-8. doi: 10.1056/NEJM197410102911503. PMID: 4414996.
8. McLaughlin VV, Archer SL, Badesch DB, Barst RJ, Farber HW, Lindner JR, Mathier MA, McGoon MD, Park MH, Rosenson RS, Rubin LJ, Tapson VF, Varga J, Harrington RA, Anderson JL, Bates ER, Bridges CR, Eisenberg MJ, Ferrari VA, Grines CL, Hlatky MA, Jacobs AK, Kaul S, Lichtenberg RC, Lindner JR, Moliterno DJ, Mukherjee D, Pohost GM, Rosenson RS, Schofield RS, Shubrooks SJ, Stein JH, Tracy CM, Weitz HH, Wesley DJ; ACCF/AHA. ACCF/AHA 2009 expert consensus document on pulmonary hypertension: a report of the American College of Cardiology Foundation Task Force on Expert Consensus Documents and the American Heart Association: developed in collaboration with the American College of Chest Physicians, American Thoracic Society, Inc., and the Pulmonary Hypertension Association. Circulation. 2009 Apr 28;119(16):2250-94. doi: 10.1161/CIRCULATIONAHA.109.192230. Epub 2009 Mar 30. Erratum in: Circulation. 2009 Jul 14;120(2):e13. PMID: 19332472.
9. Saggar R, Ross DJ, Saggar R, et al. Pulmonary hypertension associated with lung transplantation obliterative bronchiolitis and vascular remodeling of the allograft. Am J Transplant. 2008;8(9):1921-1930. doi:10.1111/j.1600-6143.2008.02338.x
10. Levine DJ, Glanville AR, Aboyoun C, Belperio J, Benden C, Berry GJ, Hachem R, Hayes D Jr, Neil D, Reinsmoen NL, Snyder LD, Sweet S, Tyan D, Verleden G, Westall G, Yusen RD, Zamora M, Zeevi A. Antibody-mediated rejection of the lung: A consensus report of the International Society for Heart and Lung Transplantation. J Heart Lung Transplant. 2016 Apr;35(4):397-406. doi: 10.1016/j.healun.2016.01.1223. Epub 2016 Feb 10. PMID: 27044531.
11. Popper HH, Juettner-Smolle FM, Pongratz MG. Micronodular hyperplasia of type II pneumocytes--a new lung lesion associated with tuberous sclerosis. Histopathology. 1991 Apr;18(4):347-54. doi: 10.1111/j.1365-2559.1991.tb00856.x. PMID: 2071093.
12. Hogg JC. Lung structure and function in COPD. Int J Tuberc Lung Dis. 2008 May;12(5):467-79. PMID: 18419881.
13. Rossi G, Cavazza A, Spagnolo P, Bellafiore S, Kuhn E, Carassai P, Caramanico L, Montanari G, Cappiello G, Andreani A, Bono F, Nannini N. The role of macrophages in interstitial lung diseases: Number 3 in the Series "Pathology for the clinician" Edited by Peter Dorfmüller and Alberto Cavazza. Eur Respir Rev. 2017 Jul 19;26(145):170009. doi: 10.1183/16000617.0009-2017. PMID: 28724562.
14. Flaherty KR, Colby TV, Travis WD, Toews GB, Mumford J, Murray S, Thannickal VJ, Kazerooni EA, Gross BH, Lynch JP 3rd, Martinez FJ. Fibroblastic foci in usual interstitial pneumonia: idiopathic versus collagen vascular disease. Am J Respir Crit Care Med. 2003 May 15;167(10):1410-5. doi: 10.1164/rccm.200204-373OC. Epub 2003 Feb 20. PMID: 12615630.
15. Epler GR, Colby TV, McLoud TC, Carrington CB, Gaensler EA. Bronchiolitis obliterans organizing pneumonia. N Engl J Med. 1985 Jan 17;312(3):152-8. doi: 10.1056/NEJM198501173120304. PMID: 3965933.
16. Beasley MB, Franks TJ, Galvin JR, Gochuico B, Travis WD. Acute fibrinous and organizing pneumonia: a histological pattern of lung injury and possible variant of diffuse alveolar damage. Arch Pathol Lab Med. 2002 Sep;126(9):1064-70. doi: 10.5858/2002-126-1064-AFAOP. PMID: 12204055.
17. Katzenstein AL, Bloor CM, Leibow AA. Diffuse alveolar damage--the role of oxygen, shock, and related factors. A review. Am J Pathol. 1976 Oct;85(1):209-28. PMID: 788524; PMCID: PMC2032554.

18. Ashcroft T, Simpson JM, Timbrell V. Simple method of estimating severity of pulmonary fibrosis on a numerical scale. J Clin Pathol. 1988;41(4):467-470. doi:10.1136/jcp.41.4.467
19. Ofek E, Sato M, Saito T, Wagnetz U, Roberts HC, Chaparro C, Waddell TK, Singer LG, Hutcheon MA, Keshavjee S, Hwang DM. Restrictive allograft syndrome post lung transplantation is characterized by pleuroparenchymal fibroelastosis. Mod Pathol. 2013 Mar;26(3):350-6. doi: 10.1038/modpathol.2012.171. Epub 2012 Sep 28. PMID: 23018877.
20. Katzenstein AL, Zisman DA, Litzky LA, Nguyen BT, Kotloff RM. Usual interstitial pneumonia: histologic study of biopsy and explant specimens. Am J Surg Pathol. 2002 Dec;26(12):1567-77. doi: 10.1097/00000478-200212000-00004. PMID: 12459623.
21. Katzenstein AL, Fiorelli RF. Nonspecific interstitial pneumonia/fibrosis. Histologic features and clinical significance. Am J Surg Pathol. 1994 Feb;18(2):136-47. PMID: 8291652.

Table S1. Overview of the histological scoring grid that was used to assess BOS explant lungs. All parameters were assessed and scored as absent/present. BOS: bronchiolitis obliterans syndrome; AR: acute rejection; AFOP: acute fibrinous and organizing pneumonia; RBCs: red blood cells; OP: organizing pneumonia; N: number; PPFE: pleuroparenchymal fibro-elastosis; UIP: usual interstitial pneumonia; NSIP: non-specific interstitial pneumonia.

**Table S2.** Comparison of patient characteristics between BOS and non-CLAD patients			
	**BOS**	**Non-CLAD**	**p value**
**Patients, N**	52	26	
**Age at transplant (y)**	43 (27-57)	58 (43-62)	**0.0025**
**Male, N (%)**	21 (40)	17 (65)	0.054
**Underlying disease, N (%)**			0.18
Emphysema	17 (33)	9 (35)	
ILD	10 (19)	9 (35)	
CF + BRECT	19 (37)	3 (12)	
PHT + Eisenmenger	4 (8)	3 (12)	
Other	2 (4)	2 (8)	
**Type of transplant, N (%)**			0.98
SSLTx	37 (71)	19 (73)	
HLTx	4 (8)	2 (8)	
SLTx	11 (21)	5 (19)	
**Time to graft loss (y)**	5.4 (2.3 – 8.2)	1.4 (0.7-3.2)	**0.0036**
**Post-LTx best FEV_1 (l)**	2.4 (1.9 – 3.1)	2.5 (1.9 – 3.1)	0.84
**Post-LTx best FVC (l)**	3.3 (2.6 – 4.0)	3.2 (2.3 – 3.8)	0.42
**FEV_1 before graft loss (l)**	0.6 (0.5 – 0.9)	2.3 (1.7-2.7)	**<0.0001**
**FEV_1 decline (%)**	72.1 (60.8 – 79.8)	9.8 (4.5 – 14.3)	**<0.0001**
**Time last FEV1 to graft loss (d)**	29 (10 – 47)	40 (23-57)	0.36
**Ever AR, N(%)**	30 (58)	7 (27)	**0.016**
Ever severe AR (≥A2), N(%)	16 (31)	4 (15)	0.18
**Ever LB, N(%)**	17 (33)	6 (23)	0.44
Ever severe LB (=B2R), N(%)	9 (17)	1 (4)	0.15

Table S2. Comparison of patient characteristics between BOS patients and non-CLAD patients. Data are shown as n, n(%), and median (interquartile range). LTx: lung transplantation; ILD: interstitial lung disease; CF: cystic fibrosis; BRECT: bronchiectasis; PHT: pulmonary hypertension; SSLTx: sequential single-lung transplantation; HLTx: heart-lung transplantation; SLTx: single-lung transplantation; CLAD: chronic lung allograft dysfunction; BOS: bronchiolitis obliterans syndrome; AR: acute rejection; LB: lymphocytic bronchiolitis; BAL: broncho-alveolar lavage.

Table S3. Histological findings in BOS lungs stratified per transplant era

	BOS total	BOS 1991-1999	BOS 2000-2009	BOS 2010-2018	Unadjusted P value
**Patients, N**	52	9	34	9	
**Airway lesions**					
Small-airway lymphocytic bronchiolitis	20 (38)	2 (22)	15 (44)	3 (33)	0.46
Large-airway lymphocytic bronchitis	29 (56)	6 (67)	18 (53)	5 (56)	0.76
Bronchiolitis obliterans	38 (73)	5 (56)	27 (79)	6 (67)	0.32
Vanishing airways	13 (25)	3 (33)	7 (21)	3 (33)	0.60
Bronchiectasis	10 (19)	1 (11)	8 (24)	1 (11)	0.56
Follicular bronchiolitis	1 (2)	0	1 (3)	0	0.76
Mucus plugs	19 (37)	1 (11)	15 (44)	3 (33)	0.18
Squamous metaplasia	9 (17)	1 (11)	6 (18)	2 (22)	0.82
Respiratory bronchiolitis	2 (4)	1 (11)	1 (3)	0	0.42
**Vascular lesions**					
Acute rejection	8 (15)	3 (33)	5 (15)	0	0.14
Pulmonary arteriopathy	17 (33)	2 (22)	12 (35)	3 (33)	0.76
Media hypertrophy	10 (19)	1 (11)	7 (21)	2 (22)	0.79
Intima hyperplasia	17 (33)	2 (22)	12 (35)	3 (33)	0.76
Pulmonary venopathy	14 (27)	2 (22)	9 (26)	3 (33)	0.70
Intima hyperplasia	14 (27)				
Venous occlusion	5 (10)	1 (11)	2 (6)	2 (22)	0.33
Bronchial arteriopathy	11 (21)	3 (33)	8 (24)	0	0.19
Media hypertrophy	11 (21)				
Intima hyperplasia	11 (21)				
Microvascular injury	2 (4)	0	2 (6)	0	0.58
Thrombi	3 (6)	0	3 (9)	0	0.43
**Alveolar compartment**					
Pneumocyte hyperplasia	15 (29)	3 (33)	12 (35)	0	0.11
Emphysema	29 (56)	7 (78)	18 (53)	4 (44)	0.31
Hemosiderophages	15 (29)	2 (22)	11(32)	2 (22)	0.75
Neutrophils in alveoli	7 (13)	2 (22)	4 (12)	1 (11)	0.70
Eosinophils in alveoli	1 (2)	0	1 (3)	0	0.76
Organizing pneumonia	11 (21)	1 (11)	7 (21)	3 (33)	0.51
AFOP	4 (8)	0	3 (9)	1 (11)	0.62
Hyaline membranes	5 (10)	1 (11)	3 (9)	1 (11)	0.97

Cholesterol clefts	8 (15)	2 (22)	5 (15)	1 (11)	0.79
Giant cells	13 (25)	3 (33)	10 (29)	0	0.16
RBCs intra-alveolar	27 (52)	5 (56)	18 (53)	4 (44)	0.88
Fibrin intra-alveolar	13 (25)	2 (22)	10 (29)	1 (11)	0.52
Foamy macrophages intra-alveolar	10 (19)	0	8 (24)	2 (22)	0.27
**Fibrotic lesions**					
Ashcroft score ≥5	28 (54)	4 (44)	19 (56)	5 (56)	0.82
Bronchocentric fibrosis	21 (40)	3 (33)	15 (44)	3 (33)	0.75
Paraseptal fibrosis	17 (33)	3 (33)	12 (35)	2 (22)	0.76
Subpleural fibrosis	15 (29)	3 (33)	9 (26)	3 (33)	0.87

Table S3. Comparison of histological findings in BOS explant lungs stratified per lung transplant era. Data are shown as n and n(%). BOS: bronchiolitis obliterans syndrome; AFOP: acute fibrinous and organizing pneumonia; RBC: red blood cells.

References supplements

1. Vanstapel A, Verleden SE, Weynand B, et al. Late-onset "acute fibrinous and organising pneumonia" impairs long-term lung allograft function and survival. Eur Respir J. 2020. ;56(3):1902292.

2. Verleden GM, Glanville AR, Lease ED, et al. Chronic lung allograft dysfunction: Definition, diagnostic criteria, and approaches to treatment—A consensus report from the Pulmonary Council of the ISHLT. J Heart Lung Transplant. 2019 ;38(5):493-503.

3. Glanville AR, Verleden GM, Todd JL, et al. Chronic lung allograft dysfunction: Definition and update of restrictive allograft syndrome—A consensus report from the Pulmonary Council of the ISHLT. J Hear Lung Transplant. 2019 ;38(5):483-492.

4. Ashcroft T, Simpson JM, Timbrell V. Simple method of estimating severity of pulmonary fibrosis on a numerical scale. J Clin Pathol. 1988;41(4):467-470.
